# Improving teamwork between students from two professional programmes in dental education

**DOI:** 10.1111/j.1600-0579.2011.00702.x

**Published:** 2012-02

**Authors:** L Leisnert, M Karlsson, I Franklin, L Lindh, K Wretlind

**Affiliations:** Faculty of Odontology, Malmo UniversityMalmo, Sweden

**Keywords:** teamwork, undergraduate dental education, intervention

## Abstract

In Sweden, the National Board of Health and Welfare forecasts a decrease in dentists with 26% and an increase in dental hygienists with 47% until the year of 2023. This, together with changes in both epidemiology, especially of dental caries, and political priorities, calls for an effective and well-developed cooperation between dentists and dental hygienists in future dentistry. Hence, the aim of this project was to investigate whether highlighting teamwork during the undergraduate studies of dental students and dental hygiene students could improve the students’ holistic view on patients as well as their knowledge of and insight into each other’s future professions. Thirty-four dental students and 24 dental hygiene students participated in the study. At the beginning of their final year in undergraduate education, a questionnaire testing the level of knowledge of the dental hygienists’ clinical competences was completed by both groups of students. In addition, activities intending to improve teamwork quality included the following: (i) a seminar with a dentist representing the Public Dental Health Services in Sweden, (ii) dental students as supervisors for dental hygiene students, (iii) planning and treatment for shared patients and (iv) students’ presentations of the treatments and their outcomes at a final seminar. The project was ended by the students answering the above-mentioned questionnaire for the second time, followed by an evaluation of the different activities included in the study. The knowledge of dental hygienists’ competences showed higher scores in almost all questions. Both groups of students considered the following aspects important: seminars with external participants, dental students acting as supervisors and planning and treating shared patients. By initiating and encouraging teamwork between dental students and dental hygiene students, it is possible to increase knowledge on dental hygienists’ competence and also to develop and strengthen a holistic view on patients and dental work, thereby preparing both groups of students for their professional life.

## Introduction

Epidemiological changes in dental diseases, ageing populations with an increasing need of complex dental treatment and the changed political priority to funding in dentistry underline the needs of improved cooperation between the different members of the dental team. Furthermore, the ratio of dentists to dental hygienists is forecasted to change in some countries. In Sweden, the number of dentists is forecasted to decrease with 26%, landing on around 5400 dentists in 2023 (1), while dental hygienists are expected to increase by 47% to 4700 (1). Although the dental teams vary in structure and composition between different countries, general changes in the surrounding society demand flexibility and proper knowledge on competencies for each professional group (2) to rise to these mounting challenges.

Parallell to these changes, the discussion of the formal and informal competences of dental hygienists, and in extension their role within dentistry, has been going on for decades. Still, after more than 30 years of cooperation between the groups, articles such as ‘Who does what and why’? (3) are published in dental magazines, this one in 2008. Four years prior to this, the same magazine ran ‘Cost effective teamwork’ (4) as the editorial, in which it was stated that ‘until recently many dentists were still slightly uncomfortable about other people actually treating their patients’. This debate has taken place in most European countries in which dental hygienists have become a part of the oral health system. Klefbom et al. (5) conclude that dentists’ knowledge on the competences of dental hygienists ought to be improved to facilitate teamwork. They also suggest that cooperation and integration in undergraduate education could be a way to enhance knowledge on respective professions’ competencies.

Traditionally, the education of dentists and dental hygienists has taken place as uniprofessional educations where students learn in isolation from each other (6), but growing evidence supporting the idea that interprofessional education (IPE) will improve abilities both to work as a team and to communicate more effectively with colleagues and patients (7) must also be taken into account. However, most of the research regarding IPE within the medical field concerns the relationship between doctors and nurses, and only a few papers are concerned with dentistry (8). In a survey of IPE, including seven academic health centres (8) that have schools of dentistry associated with them, a review was made and completed with interviews. One conclusion was that dental schools were isolated from other schools and not interested in IPE. Another conclusion pointed to the importance of dental schools becoming an active participant in future interprofessional educational initiative. Furthermore, in a study examining how teamwork influences resource planning in acute hospitals, it was concluded that effective teamwork is one of the important factors to the success of discharge planning. (9).

Thus, a general agreement has emerged that improving teamwork is important for achieving both better and more cost-effective treatment for patients (3, 7, 8).

### Objectives

This study had the objective of examining whether placing a stronger emphasis on teamwork during the undergraduate studies of dental students and dental hygiene students could:

- Increase knowledge of and insight into the respective future professions with special emphasis on the dental hygienists field of competence.
- Develop the holistic view and approach towards patients, as experienced by the students.

## Material and methods

Students from two dental programmes, dental hygiene students and dental students, participated in the study.

### Educational context of the dental programmes in Malmö

The dental and the dental hygiene programmes are taught at the Faculty of Odontology at Malmö University, and they are guided by four linked principles: (i) Self-directed learning, (ii) Holistic view of patient care, (iii) Oral health and (iv) Teamwork. Self-directed learning is implemented as problem-based learning throughout the programmes (10). The holistic view is interpreted as caring for the individual rather than as producing quantities of items of dental treatment. Such an approach towards the patient should encourage students to use their knowledge and understanding, skills and ability, judgement and stance as expressed in the Swedish Higher Education Ordinance (11). In turn, the holistic view provides a platform for *oral health* that has been chosen over dentistry. Teamwork is developed through work in study groups and clinical settings.

In the clinical setting, students from both programmes respectively care for their own patients from their second to their last semester – for dental students their tenth and for dental hygiene students their fourth. Students have a gradually increasing responsibility for the oral health care of their patients, who require oral health needs of increasing complexity. From the onset, an environment is created in which provision of care is related to (i) a fundamental understanding of the needs of the individual patient, (ii) an evidence-based approach for the outcome of clinical interventions and (iii) an interdisciplinary approach to oral health care. Both groups of students experience an increase in intensity and opportunities for a mixed and varied care of more and more complex patients during their final semesters, and dental students assisted by dental nurses, experience comprehensive care in cooperation with dental hygiene students (12). Both dental students and dental hygiene students practise in the Public Dental Health Services (PDHS) (13).

The project was designed as an intervention study with different activities, including seminars, treating patients together and presentations of the outcomes of the treatments, framed by pre- and post-test. As a pre-test, we used a questionnaire mapping the students’ knowledge on a sample of the dental hygienists competencies. Post-test included answering the same questionnaire once more, with questions relating to how the different activities were experienced and to what extent they were deemed useful by the students.

### Project organisation

In the research group for the project, responsible for planning, directing and carrying out the activities, students and staff from both the dental hygiene and dental programmes participated. The project was introduced and started during the spring 2007 by launching a website within the learning management system of the university, acting as a platform for both information and interactions.

### Participants

Beginning from their eighth and second semester respectively, 34 dental students and 24 dental hygiene students participated in the study. The number of students corresponded to the size of the courses. Teams consisting of one dental student and one dental hygiene student were formed. As the number of dental students was greater than that of dental hygiene students, some dental hygiene students had two dental students to cooperate with.

### Activities

**Fig 1:**
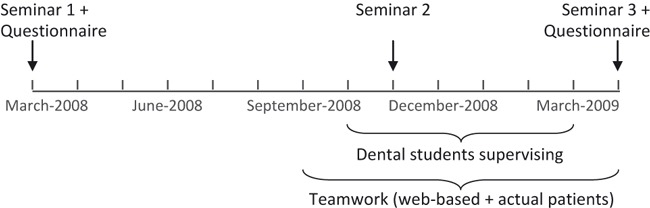
Timetable for performed activities.

The timetable for included activities is shown in Fig. 1.

#### Questionnaires.

At the start of the project, 23 dental hygiene students (*N* = 24) and 32 dental students (*N* = 34) answered a questionnaire (7), Appendix 1, at the same occasion. The questionnaire consisted of 23 questions on whether or not dental hygienists are licensed for the competences described in the different questions. The same questionnaire was answered 1 year later. Between the two tests a number of activities were performed.

#### Seminars

Three seminars were held during the course of the study:

Seminar 1: An introduction of the project including a session with a dentist from the PDHS, presenting the visions and experiences of a teamwork model developed and successfully practised at the PDHS.

Seminar 2: Presentations held by ten chosen teams of students, each group presenting different aspects of how to plan and carry out the treatment for two web-based patients. All students attended this seminar together with supervisors from different fields of Odontology.

Seminar 3: Presentations given by six chosen teams of students, each group presenting how they planned and carried out the treatment for shared patients in the students’ clinic. Discussions on the outcome of said treatment were also an integral part of this seminar.

Prior to the final session, the students were asked to write down problems and possibilities that they had encountered during their team collaborations. These were discussed during the last seminar, as well as the students’ suggestions for future professional cooperation.

#### Teamwork

As mentioned earlier, the dental students and the dental hygiene students were divided into teams. In these teams, they planned and carried out the treatment for both web-based patients and actual patients attending the students′ clinic. The web-based cases were presented and made available on the website of the project, where electronic folders for each team were created as well. In the folders, the teams documented their discussions and agreements regarding the web-based cases on following items: diagnosis, treatment planning and prognosis. In the folder, they also had to document, present and discuss 2—4 shared patients from the student’s clinic. During the students’ clinical work, they and their clinical instructors could draw on one experienced dentist and on one experienced dental hygienist to support them with encouragement and pinpointing opportunities and advantages of teamwork.

#### Supervising

To increase the interaction surface between students and to an even greater extent provide opportunities for developing understanding and knowledge on how to cooperate as a dental team, the dental students supervised the dental hygiene students in their clinical practice in one to two occasions.

### Students’ opinions on different activities and parts of the project

Students assessed the different activities and how they valued their contribution in developing successful teamwork. The questionnaire, Appendix 2, used for this purpose was designed with a number of statements, concerning the different activities, where students could mark on a visual analogue scale from 1 to 10 whether they agreed or disagreed on the statements.

### Methodological considerations

The first questionnaire was answered by 32 of 34 dental students and 23 of 24 dental hygiene students. On the second occasion, 30 of 32 dental students and 20 of 20 dental hygiene students answered. The missing answers were attributable to electives, Erasmus exchange, interrupted studies or illness.

### Statistical methods

**Fig 2:**
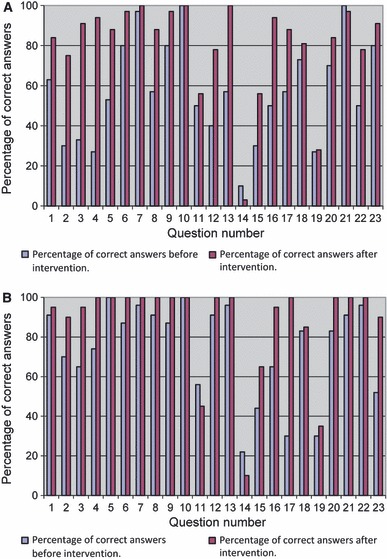
(a) Percentage of correct answers on each of the 23 questions in the questionnaire, before and after intervention. Dental students. (b) Percentage of correct answers on each of the 23 questions in the questionnaire, before and after intervention. Dental hygiene students.

**Fig 3:**
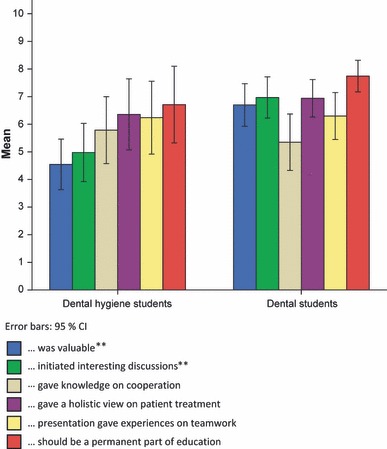
Students’ ratings of six statements regarding the fictional web-based clinical cases. **0.001 ≤ P < 0.01.

**Fig 4:**
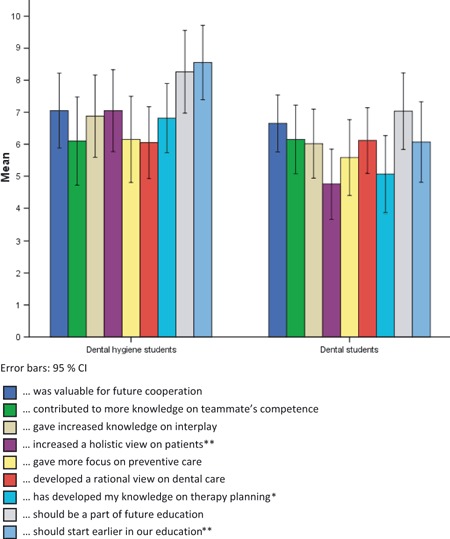
Students’ ratings of nine statements regarding dental students supervising dental hygiene students in the clinical setting. *0.01 > P < 0.05, **0.001 ≤ P < 0.01.

**Fig 5:**
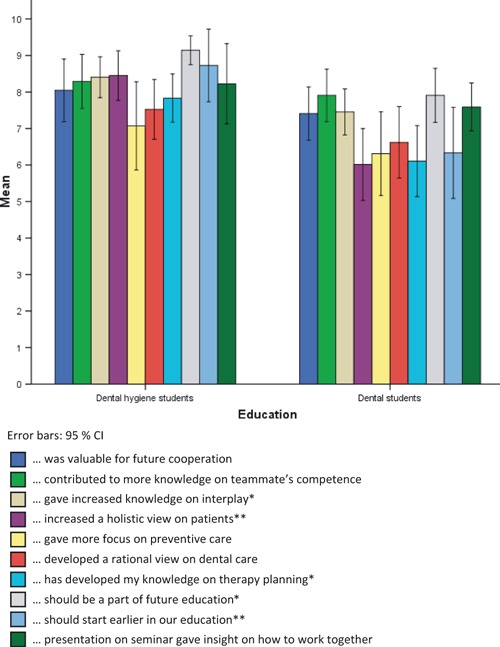
Students’ ratings of ten statements regarding teamwork with shared patients. *0.01 > P < 0.05, **0.001 ≤ P < 0.01.

Statistical comparisons of the percentage of correct answers before and after intervention, within each group, were performed using a Sign test, Figs 2a,b. Independent samples *t*-test was used in the comparisons of the two student groups in the evaluation of activities shown in Figs 3–5. The significance level in all tests was α = 5%. The program used was PASW/SPSS for Windows, release 18.0.0 2009 (SPSS Inc., Chicago, IL, USA). The significance level is marked besides respectively statements in Figs 3–5. *0.01 > *P* < 0.05, **0.001 ≤ *P* < 0.01, ***P < 0.001.

## Results

### Students’ knowledge on the competences of the dental hygienist

The results of the first questionnaire showed that it was mostly the dental student who lacked knowledge on the competences of the dental hygienist. In nine of 23 questions more than 50% provided a wrong answer. For instance, between 50% and 70% did not know that dental hygienists (in Sweden) are allowed to decide on, carry out and diagnose X rays concerning caries and periodontitis; prescribe alcohol, fluoride and anaesthetics to their place of work; decide on and carry out bleaching of teeth; glue small pieces of jewellery on the teeth; and/or decide on and carry out bacterial analyses of saliva. Concerning dental hygiene students more than 50% answered wrongly in five of the 23 questions. Their gaps of knowledge were on the following: not aware of being allowed to possess X-ray equipment when practising on their own; not being allowed to diagnose diseases in mucous membrane; being allowed to carry out fissure blocking; decide on and carry out bleaching of teeth; and/or to manufacture a bleaching tray. At the concluding seminar, the students’ knowledge had improved in almost all matters of the questionnaire. For the dental students group, there were significant changes concerning questions 2, 3, 4, 5, 8, 12, 13, 16 and 17, and for the dental hygiene students, there were significant changes concerning questions 17 and 23. (Fig. 2a,b).

### Evaluation of activities carried out during the project

#### Seminar on teamwork with dentist from PDHS.

Dental hygiene students (DHS) rated this seminar with mean score (SD) of 6.9 (2, 7), while dental students’ (DS) mean score (SD) was 5.3 (2, 3).

#### The fictional web-based clinical cases: treatment planning in teams followed by a seminar with a presentation of the suggested treatment.

A significant difference between the DHS and the dental students, on to which degree this activity was valuable or not and to what extent the seminar initiated interesting discussions, was noted.

Dental students rated this activity lower in regard to its contribution to improve teamwork as well as to increase the knowledge on cooperation. Nevertheless, both groups found it valuable to make this part of education permanent with mean scores (SD) for DHS 6.7 (2.9) and DS 7.7 (1, 6) (Fig. 3).

#### Have the two questionnaires on the competences of dental hygienists increased your knowledge on which competences dental hygienists have?

Dental hygiene students gave mean score (SD) 6.6 (2.7) and dental students 7.2 (2.4).

#### Dental students supervising DHS.

This part scored high especially among the DHS who claimed that the teamwork had increased, that the holistic approach on patients had been strengthened with mean score (SD) of 7.1 (2, 7) and that they had gained valuable experiences for future cooperation. Concerning the holistic view on patients, there was a significant lower mean score (SD) from the dental students 4.8 (2.9). Other significant differences were that DHS felt that this moment should start earlier in education and that it should develop their knowledge on therapy planning. Both groups found it valuable to make this part permanent with mean scores (SD) DHS 8.2 (2.7) and DS 7.0 (2.5) (Fig. 4).

#### Teamwork with shared patient.

Both groups of students felt that treating shared patients should become a permanent part of the education with mean scores (SD) DHS 9.1 (0.9) and DS = 7.9 (1.5). There were significant differences between the two groups in five of the questions. It concerned the questions whether this moment gave increased knowledge on interplay, increased holistic view on patients, has developed knowledge on therapy planning and should be a part of future education, and that the presentations on the seminar gave insight into how to work together. The questions display the same pattern of DHS giving higher scores. (Fig. 5).

## Discussion

### Knowledge on competence

Both DHS and dental students in this project demonstrate an increase in knowledge regarding the competences of dental hygienists as well as a perceived increased understanding and appreciation of some of the common principles of the programmes. Moreover, according to what the students expressed in the evaluation comments, merely sharing patients, planning and performing treatment together, contributed to a more holistic view of the patient and gave valuable experiences for cooperation in their future professional roles. Thus, without this cooperation, especially the dental students would have graduated with considerable knowledge gaps of the dental hygienists’ competences and it would presumably have proven to be an obstacle in developing a fruitful and effective teamwork.

The reasons behind testing only the students’ knowledge on competences of the dental hygienist were several. First, Klefbom et al. (5) found that dentists’ knowledge on this issue was low; further, they suggested that undergraduate dental students should be better prepared within this field to be able to lead and develop a successful teamwork. Second, the competences of dentists are not limited like those of the dental hygienist and are therefore more clear cut and known – we felt no need to ask for them. Third, the development of teamwork between DHS and dental students depends to a large degree on the students’ knowledge of what a dental hygienist license includes.

### Seminar with dentist from PDHS

The relatively low ratings on the seminar with a dentist from the PDHS could be an effect of a poor performance of the lecturer because the comments on the event reveal that it was valued positive in terms of initiating reflections and that the seminar was stated as a good start for the project. The seminars with participants from dental care external to the faculty were recommended to be a permanent part of the curriculum in that it could stimulate students’ knowledge and reflections on how to perform good dentistry.

### Web-based clinical cases

In general, the dental students were more positive to this activity. Judging from the written comments, the reason behind this is that the cases consisted of complicated prosthetic cases where the dental students were more familiar with compared to the dental hygienist students. Comments suggest that the DHS found it too difficult to participate in the debate as the case was too ‘dentist’ orientated.

### The two questionnaires

When asked if the two questionnaires contributed to increased knowledge, the dental students gave higher scores, indicating that they felt their knowledge had increased more. It is likely that this is because of their initial great gaps and thus had more to learn.

### Dental students supervising DHS and cooperation with shared patients

It is harder to grasp why DHS to a higher degree than dental students claimed that they received a more holistic view on patients when treating shared patients and/or when dental students acted as supervisors. One explanation could be that the DHS experienced broader perspectives and more complex approaches towards treatment planning and actual treatment when working together with dental students than vice versa. This is perhaps also illustrated by the fact that the comments from the DHS were more numerous and overall positive, while the dental students offered very few. Another explanation could be that the dental students already had a developed holistic view and therefore felt that they were less likely to increase it to a significant extent. However, our overall impression is that dental students acting as supervisors for DHS contribute to developing DHS’ ability to acquire a more holistic view on patient care, and the fact that both student groups recommended this activity to become a permanent part of the education also indicates that dental students valued it.

### Possible errors of the study

As mentioned earlier, there were 34 dental students and 24 DHS participating in the study, which resulted in that some dental students cooperated with two DHS instead of one. As the questionnaires were answered anonymously, we cannot separate results from DHS that worked with one or two dental students. It would have been relevant to see whether this could have any impact on the results, but it is not possible.

## Conclusions

By initiating teamwork between dental students and DHS during their undergraduate education, it was possible to:

- increase students’ knowledge on dental hygienists competence
- develop students’ perceived holistic view on patients

## Appendix 1 Questionnaire regarding the competences of the dental hygienist

**Table:**

	Yes	No	Do not know
A licensed dental hygienist is entitled to….	□	□	□
1. Offer and give care in an office of her/his own	□	□	□
2. Possess a radiography equipment in her/his office	□	□	□
3. Decide on and perform radiological examination	□	□	□
4. Radio graphically diagnose carious lesions	□	□	□
5. Clinically diagnose carious lesions (by aid of mirror and explorer)	□	□	□
6. Radio graphically diagnose periodontitis	□	□	□
7. Clinically diagnose periodontitis by aid of mirror and pocket probe	□	□	□
8. Assess risks for the reoccurrence of caries and plan for preventive measures	□	□	□
9. Assess risks for the reoccurrence of periodontitis and plan for preventive measures	□	□	□
10. Inject local anaesthetics	□	□	□
11. Independently procure local anaesthetics	□	□	□
12. Prescribe fluorides for treatment of carious lesions	□	□	□
13. Seal fissures (apply resins on teeth without preparation, to avoid carious lesions)	□	□	□
14. Block fissures (apply resins on teeth after minimal preparation, (to avoid carious lesions)	□	□	□
15. Plan for and perform tooth bleaching	□	□	□
16. Plan for and stick ‘tooth jewellery’ on teeth	□	□	□
17. Take impression for and produce appliances for local application of fluoride and/or tooth bleaching	□	□	□
18. Place restorations after tooth preparations made by a dentist	□	□	□
19. Decide whether cervical tooth substance losses (due to too heavy tooth brushing) should be treated and subsequently place the restoration	□	□	□
20. Plan for and perform preventive measures in outreach activities among patients in special need of care	□	□	□
21. Plan for and perform information on oral health measures in schools and nurseries	□	□	□
22. Decide on and perform intra oral bacterial sampling	□	□	□
23. Examine and diagnose diseases in the oral mucosa	□	□	□

## Appendix 2 Evaluation of team-work between dental students and dental hygiene students spring 2007—spring 2008

I am □ a dental student □ dental hygienist student.

**Table:**

**1. Seminar on team-work – Dr Eva Åberg from Ängelholm, DPHS Region of Skåne, presented a model for team-work: dentist – dental hygienist – dental nurse**
What did you gain from this seminar (e.g. inspiration, insight, got goose bumps, got bored, reflection, commitment, reluctance)?
This moment belongs to those that should be permanent in the education!	**Mark on the line to what extent this statement in your opinion is correct**	
**Not at al correct**	**Totally correct**
If not correct – how do you suggest it to be improved? ....

**Table:**

**2. Patient cases presented on webzone – co-written treatment plans on the web followed by seminar with presentations from selected teams**
**The moment with team-work on patient cases on Webzone:**	**Mark on the lines to what extent this statement in your opinion is correct**	
**Not at all correct**	**Totally correct**
The selected presentations of shared patients was of substantial value	———————————————————	
Comment: Why/ why not?		
The selected presentations of shared patients gave rise to valuable discussions	———————————————————	
Comment: ...		
The selected presentations of shared patients gave insight in what collaboration means	———————————————————	
Comment: ...		
The selected presentations of shared patients gave rise to a holistic view on the patient	———————————————————	
Comment:		
The selected presentations of shared patients in a seminar gave an insight in what a collaboration might imply	———————————————————	
Comment: ...		
What did you gain from this seminar (e.g. inspiration, insight, got goose bumps, got bored, reflection, commitment, reluctance)? . . . .
This moment belongs to those that should be permanently included in the education!	**Not at all correct**	**Totally correct**
———————————————————	
If not correct – how do you suggest it to be improved? . . . .

**Table:**

**3. Questionnaires**	**Mark on the line to what extent this statement in your opinion is correct**	
The questionnaires on the competences of a dental hygienist have improved my knowledge on dental hygienist/dentist competence	**Not at all correct**	**Totally correct**
———————————————————	

**Table:**

**4. The supervision by the dental students in the clinic**
	**Mark on the line to what extent the statement in your opinion is correct**	
	**Not at all correct**	**Totally correct**
———————————————————	
Has provided a base for future collaboration	———————————————————	
Comment: ....		
Has contributed to a better understanding of the team-member competence	———————————————————	
Comment:		
Has contributed to a better understanding of how the team-work between a dentist and a dental hygienist might work	———————————————————	
Comment:		
Has contributed to an improved holistic view on the patient	———————————————————	
Comment:		
Has created a base for more focus on prevention and causal treatment	———————————————————	
Comment:		
Has contributed to the development of my opinion on rational dental work	———————————————————	
Comment:		
Has in combination with the treatment planning for these patients developed my knowledge	———————————————————	
Should in the future be a mandatory part of the education	———————————————————	
Comment:		
Should start earlier	———————————————————	
Comment:		
**For dental students:**		
The instruction for the supervision was relevant and sufficient	———————————————————	
The instruction facilitated the supervision!	———————————————————	

**Table:**

**5.Collaboration with shared patients from spring 2007—spring 2008**
	**Mark on the line etc…**	
**The moment with shared patients:**	**Not at all correct**	**Totally correct**
Has provided a base for future team-work	———————————————————	
Comment:		
Has contributed to a better understanding of the team-member’s competence	———————————————————	
Comment:		
has contributed to a better understanding of how the collaboration between a dentist and a dental hygienist works	———————————————————	
Comment:		
Has contributed to an improved holistic view on the patient	———————————————————	
Comment:		
Has created a base for more focus on prevention and causal treatment	———————————————————	
Comment:		
Has contributed to the development of my opinion on rational dental work	———————————————————	
Comment:		
Has in combination with the treatment planning from these patients developed my knowledge?	———————————————————	
Comment:		
Should in the future be mandatory part of the education	———————————————————	
Comment:		
Should start earlier	———————————————————	
Comment:		
The presentation of some shared patients in a seminar gave an insight in what collaboration can imply	———————————————————	
Comment:		
What did you gain from this last seminar (e.g. inspiration, insight, got goose bumps, got bored, reflection, commitment, reluctance)?
